# Anti-Breast Cancer Potential of Quercetin via the Akt/AMPK/Mammalian Target of Rapamycin (mTOR) Signaling Cascade

**DOI:** 10.1371/journal.pone.0157251

**Published:** 2016-06-10

**Authors:** Amilcar Rivera Rivera, Linette Castillo-Pichardo, Yamil Gerena, Suranganie Dharmawardhane

**Affiliations:** 1 Department of Biochemistry, School of Medicine, University of Puerto Rico Medical Sciences Campus, San Juan, Puerto Rico; 2 Department of Pathology and Laboratory Medicine, Universidad Central del Caribe, Bayamón, Puerto Rico; 3 Department of Pharmacology and Toxicology, University of Puerto Rico Medical Sciences Campus, San Juan, Puerto Rico; University of South Alabama, UNITED STATES

## Abstract

The Akt/adenosine monophosphate protein kinase (AMPK)/mammalian target of rapamycin (mTOR) pathway has emerged as a critical signaling nexus for regulating cellular metabolism, energy homeostasis, and cell growth. Thus, dysregulation of this pathway contributes to the development of metabolic disorders such as obesity, type 2diabetes, and cancer. We previously reported that a combination of grape polyphenols (resveratrol, quercetin and catechin: RQC), at equimolar concentrations, reduces breast cancer (BC) growth and metastasis in nude mice, and inhibits Akt and mTOR activities and activates AMPK, an endogenous inhibitor of mTOR, in metastatic BC cells. The objective of the present study was to determine the contribution of individual polyphenols to the effect of combined RQC on mTOR signaling. Metastatic BC cells were treated with RQC individually or in combination, at various concentrations, and the activities (phosphorylation) of AMPK, Akt, and the mTOR downstream effectors, p70S6 kinase (p70S6K) and 4E binding protein (4EBP1), were determined by Western blot. Results show that quercetin was the most effective compound for Akt/mTOR inhibition. Treatment with quercetin at 15μM had a similar effect as the RQC combination in the inhibition of BC cell proliferation, apoptosis, and migration. However, cell cycle analysis showed that the RQC treatment arrested BC cells in the G1 phase, while quercetin arrested the cell cycle in G2/M. *In vivo* experiments, using SCID mice with implanted tumors from metastatic BC cells, demonstrated that administration of quercetin at 15mg/kg body weight resulted in a ~70% reduction in tumor growth. In conclusion, quercetin appears to be a viable grape polyphenol for future development as an anti BC therapeutic.

## Introduction

Metastasis remains a major cause of death from breast cancer (BC), and it is estimated that 20–50% of patients diagnosed with primary mammary tumors will eventually develop metastasis [1]. The phosphoinositide 3-kinase (PI3-K)/Akt/mammalian target of rapamycin (mTOR) pathway has been specifically associated with metastasis [2]. Therefore, this pathway is highly relevant for targeted therapies for metastatic cancers, including BC.

The PI3-KAkt/mTOR pathway plays a central role in regulating protein synthesis and cell proliferation, and is associated with tumorigenesis, angiogenesis, tumor growth, and metastasis [2,3]. The serine/threonine kinase Akt (protein kinase B) is the central mediator of the PI3-K pathway with multiple downstream effectors that influence key cellular processes. Akt is activated by phosphorylation at thr^308^ by the PI3-K regulated phospholipid dependent kinase (PDK)1, and at ser^473^ by the mTOR Complex 2 (mTORC2), which results in maximal activation. Once activated, Akt regulates various cellular functions including cell metabolism, protein synthesis, inhibition of apoptosis, cell-cycle progression, induction of epithelial to mesenchymal transition, and migration/invasion. Consequently, hyperactivation of Akt and the PI3K signaling pathway in a number of human tumors has been related to advanced disease and poor prognosis [2,4]. In BC, approximately 20–55% of patients exhibit Akt hyperactivation; thus, highlighting a role for Akt as a therapeutic target [5].

Akt regulates protein synthesis and cell growth by activating mTOR, an atypical serine/threonine protein kinase that belongs to the PI3K-related kinase family and interacts with several proteins to form two distinct complexes named mTORC1 and mTORC2. Akt activates mTORC1 through an inhibitory phosphorylation of the intermediary tuberous sclerosis complex (TSC1/2). The activated mTORC1 directly phosphorylates the eukaryotic translation initiation factor 4E (eIF4E)-binding protein 1 (4E-BP1) and p70S6 kinase 1 (p70S6K), which in turn, promotes protein synthesis. Therefore, the mTOR pathway is highly relevant for cancer pathogenesis [2,4].

In addition to Akt, AMP-activated protein kinase (AMPK) is a major regulator of cellular energy metabolism. However, AMPK acts opposite to Akt, and is a negative regulator of the mTOR pathway, which has been correlated with tumor suppression and better prognosis in cancer patients. Approximately 90% of primary BCs show reduced AMPK activity; thus, exemplifying a tumor suppressive role for AMPK, which can be attributed to the inhibition of a number of anabolic pathways that promote cell growth, such as protein synthesis and fatty acid metabolism. AMPK is activated by an increase in the AMP/ATP ratio, and the subsequent phosphorylation at Thr172 by the tumor suppressor liver kinase B (LKB) or Calcium/calmodulin-dependent protein kinase kinase 2 (CaMKKβ). Activated AMPK blocks fatty acid synthesis by Acetyl CoA Carboxylase (ACC) phosphorylation, and inhibits protein synthesis via an activating phosphorylation of the TSC1/2 to result in the downregulation of mTOR and the translation elongation factor 2 (ef-2) [6,7].

The identification of mTORC1 as a downstream target of AMPK is of great interest, because of recent pre-clinical reports showing that metformin and the AMP analog 5-aminoimidazole-4-carboxamide ribose (AICAR), both pharmacological activators of AMPK, exhibit *in vivo* efficacy in blocking carcinogen-induced tumorigenesis and/or suppressing tumor growth in animal models [8]. Moreover, epidemiologic data of population-based cohort studies indicate a significantly reduced risk of breast cancer in patients with type 2 diabetes who are taking metformin on a long term basis, compared with those taking thiourea, suggesting metformin as a potential candidate for breast cancer prevention [9].

A number of PI3K/Akt/mTOR pathway targeted therapeutics are currently undergoing pre-clinical and clinical trials. Since inhibition of Akt or mTOR alone does not completely inhibit this pathway, dual PI3-K/mTOR or mTORC1/mTORC2 inhibitors are considered to be a viable alternative to aggressive cancer therapy. However, these therapies are hampered by the development of resistance due to feedback activation of growth factor receptor or Akt activity, as well as toxic side effects [10]. A safer alternative for sensitizing cancer therapies is the use of common dietary compounds with low toxicity that can inhibit therapy resistance pathways [11,12].

Grape polyphenols have been implicated in cancer protection in numerous studies due to antioxidant and pro-apoptotic effects as well as inhibition of a number of cancer causing molecular pathways [13]. In humans, grape consumption has been associated with reduced BC risk [14]. Moreover, grape juice constituents and grape seed extract (GSE) have been shown to reduce breast cancer initiation and tumor growth in rodent models [15–19], as well as block Akt activity [20].

The anticancer properties of grape extracts have been attributed to the polyphenols, where resveratrol, quercetin and catechin represent 70% of the total polyphenols in red wine [21–23]. We previously reported that an equimolar combination of resveratrol, quercetin and catechin (RQC) inhibits the PI3K/Akt/mTOR signaling pathway and breast cancer progression *in vitro*, and *in vivo*, and can act as a chemosensitization agent for anti epidermal growth factor receptor (EGFR) targeted therapy [11,24,25]. Of the RQC polyphenols, quercetin is the most effective inhibitor of the PI3K enzyme with an IC_50_ ≈ 3.8μM [26], when compared with resveratrol that has an IC_50_ ≈ 25μM [27]. Therefore, in this study, we tested the efficacy of individual quercetin as an inhibitor of the Akt/mTOR pathway in metastatic BC cells and in a mouse model of BC.

## Material and Methods

### Cell Culture

Human metastatic cancer cell lines MDA-MB-231 [ERα(-), ERβ(+), PR(-), Her2(-), EGFR(+)] and MDA-MB-435 [ER(-), PR(-), Her2(++), EGFR(-)] stably expressing green fluorescent protein (GFP) were used for this study (kind gift of Dr. Danny Welch, The University of Alabama at Birmingham, A.L., USA). Cells were cultured in Dulbecco’s modified Eagle’s medium (DMEM) supplemented with 10% fetal bovine serum (FBS) at 37°C in 5% CO_2_.

### Treatments

Resveratrol, quercetin, and catechin were purchased as 99% pure compounds (LKT Laboratories, St. Paul MN) and stock solutions made in dimethyl sulfoxide (DMSO) or ethanol.

### Western Blotting

Quiescent metastatic cancer cells in serum- and phenol red-free media were treated with vehicle, 1, 3, 5, 9, or 15μM resveratrol (Res), quercetin (Quer), or catechin (Cat), or with combined RQC at 3μM total (1μM each), 9μM total (3μM each), or 15μM total (5μM each) for 15 min. Cells were lysed immediately and total protein was quantified using the Precision Red protein assay kit (Cytoskeleton, Inc., Denver, C.O.). Equal total protein amounts were western blotted for total or active (phospho-Akt^Ser473^) Akt, (phospho-AMPK^Thr172^) AMPK, (phospho-p70S6K^Thr389^) p70S6K, or (phospho-4EBP-1^Thr37/46^) 4EBP-1. Protein activity following treatments was quantified as the level of the phospho-protein divided by total protein expression, relative to vehicle, using Image J analysis of the integrated density of positive bands.

### Cell Proliferation

MDA-MB-231 and MDA-MB-435 cells in 5% serum and phenol red-free media were treated with vehicle, resveratrol, quercetin, and catechin (RQC) at 5μM each or 15μM quercetin for 48h. Cells were fixed with methanol, nuclei stained with Propidium Iodide, and intact (non-apoptotic) nuclei were counted from digital images acquired using a microscope (model CKX41; Olympus America, Inc., Center Valley, P.A.). The total number of cells was quantified from 30 microscopic fields per well from experiments carried out on three separate days with each treatment in duplicate (n = 3).

### Cell Migration

Quiescent MDA-MB-231 and MDA-MB-435 (2x10^5^cells) were placed in the top well of a transwell chamber (Corning® FluoroBlock™-Cell Culture insert, 24 well 8.0μM pore size) with the bottom well containing: vehicle, or resveratrol, quercetin, and catechin (RQC) at 5μM each, or 15μM quercetin in serum- and phenol red- free media. After 8h incubation, cells that migrated through the 8μM pore membrane were fixed with methanol, nuclei stained with Propidium Iodide, and the nuclei were counted from digital images acquired using a microscope (model CKX41; Olympus America, Inc., Center Valley, P.A.) The total number of cells was quantified from 15 microscopic fields per well from experiments carried out on three separate days with each treatment in duplicate (n = 3).

### Flow Cytometry

MDA-MB-231 and MDA-MB-435 cells were seeded in 5% serum, phenol red-free media and treated with vehicle, combined RQC at 5μM each or quercetin at 15μM for 48h. Then, cells were washed in cold 1X PBS pH 7.4 and incubated in ice cold 70% ethanol at 4°C for 1h. After incubation, cells were washed with 1X PBS pH 7.4 and resuspended in PI master mix (Propidium Iodide 40μg/mL and RNAse 100μg/mL) for 30min at 37°C, and washed with 1X PBS pH 7.4 for cell cycle analysis.

For the apoptosis assay, cells were resuspended in annexin-binding buffer (10mM HEPES, 140mM NaCl, and 2.5mM CaCl^2^, pH 7.4.) at a concentration of 1x10^6^cells/mL. Then 5μL of Annexin-V fluorescein isothiocyanate (FITC) conjugate and 5μL of PI (4mg/mL) were added to 100μL of cells suspension and incubate for 15min at RT. Finally 400μL of annexin-binding buffer was added.

Cells were analyzed by flow cytometry using a four-color FACSCalibur (BD Biosciences, San Jose, CA) equipped with a 488nm argon-ion laser and a 635 nm red-diode laser. Cell size and granularity were determined on forward versus side scatter (FSC vs. SSC) dot plots. A total of 20,000 events were analyzed for each sample and list-mode files were collected using Cell Quest software 3.3 (BD Biosciences, San Jose, CA) and analyzed using the Flow Jo software vX.0.7 (BD Biosciences, San Jose, CA). The percentage of cells in G1, S, and G2/M phases were obtained from the cell cycle histograms.

### Apoptosis Assay

Apoptosis was measured by relative caspase 3/7 activity using a Caspase-Glo3/7 Luminescence Assay Kit as per manufacturer’s instructions (Promega, Corp., Madison, WI, USA). Equal numbers of MDA-MB-231 cells were incubated with vehicle or RQC at 5μM each or quercetin at 15μM for 48 or 96h. Next 100 μl of Caspase-3/7 Glo reagent was added and incubated at room temperature for 60 minutes. Caspase-3/7 activities were determined by quantifying luminescence.

### Animal Protocol

All animal studies were conducted under approved protocol #A8180114 by the University of Puerto Rico Medical Sciences Campus Institutional Animal Care and Use Committee, in accordance with the principles and procedures outlined in the NIH Guideline for the Care and Use of Laboratory Animals.

Female severe combined immunodeficiency (SCID) mice, 5 weeks old (Charles River Laboratories, Inc., Wilmington, MA) were maintained under pathogen-free conditions in Hepa-filtered cages with controlled light (12 h light and dark cycle), temperature (22–24°C), and humidity (25%). Throughout the experiment, the animals were provided autoclaved rodent diet (Tek Global, Harlan Teklad, Madison, WI) with 24.5% protein, 4.3% fat and 3.7% fiber and water ad libitum.

Mice were monitored daily by the research team and (or) Animal Resource Center (ARC) personnel, and weekly by the resident veterinarian, for behavior abnormalities, sudden weight loss, distress, skin abnormalities, pain, high tumor burden (more than 10% body weight and not exceeding 20mm in any one dimension), and euthanized if they appeared moribund. Mice that did not appear to be under discomfort and were not undergoing significant weight loss (as determined by weekly weighing) were sacrificed at a maximum of 2 months by cervical dislocation following an excess of isoflurane, as accepted by the current guidelines of the American Veterinary Medical Association (AVMA). The animals did not suffer undue pain or distress during the study. Mice were anesthetized by isoflurane inhalation (1–3% in air using inhalation chamber or nose cone, at 2 L/min) during inoculation, imaging, intraperitoneal injectoions, and prior to terminal cervical dislocation to minimize discomfort during procedures. Maximum size of tumors from vehicle treated mice before euthanasia was ~5 mm^3^.

### Animal treatments

Green fluorescent protein (GFP)-tagged MDA-MB-231 cells (~0.5 × 10^6^) in 1:1 Geltrex:serum free media were injected into the fourth right mammary fat pad of female SCID mice under isoflurane inhalation to produce orthotopic primary tumors. After tumor establishment (1 week post-inoculation), the animals were randomly divided into experimental treatment groups (N = 12). Mice with similar tumor area as quantified by integrated density of fluorescence images were selected for further study. Mice were injected intraperitonealy (i.p.) with vehicle (90% PBS, 10% ethanol) or 15 or 45mg/kg body weight (BW) quercetin 3X per week for 13 weeks. The fluorescent tumors were imaged 1X a week using the FluorVivo small animal *in vivo* imaging system (INDEC Systems, Inc., Santa Clara, CA) as in [28]. Average tumor growth was quantified using Image J, as the integrated density of fluorescence on each day of imaging as a function of the integrated density of fluorescence on the first day of treatment administration for each individual tumor and made relative to vehicle, as described in [24,29,30].

### Statistical Analysis

Data are expressed as the mean ± SEM. Statistical analyses were done using Graph Pad Prism 6 and Microsoft Excel. Differences between means were determined using student’s t-Test and ANOVA and are considered to be statistically significant at P≤0.05

## Results

### Effect of individual and combined RQC on mTOR signaling

**Fig 1:**
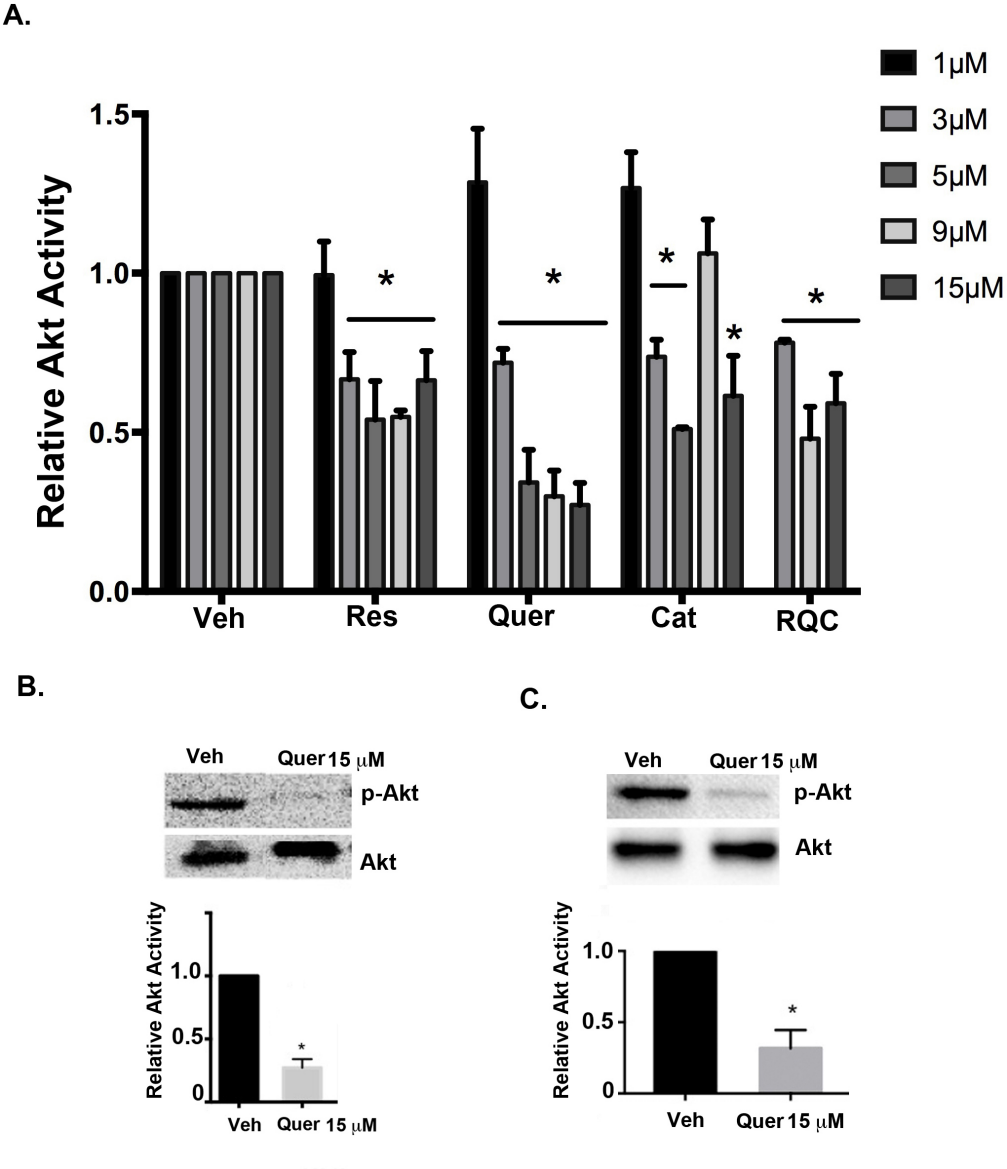
Effect of individual or combined RQC on Akt activity in breast cancer cells. Quiescent MDA-MB-231 cells were treated with vehicle, or 1, 3, 5, 9, or 15μM of resveratrol (Res), quercetin (Quer), catechin (Cat), or combined Res, Quer, and Cat (RQC) at 3μM total (1μM each), 9μM total (3μM each), or 15μM total (5μM each). MDA-MB-435 cells were treated only with vehicle or 15μM quercetin. Cells were lysed immediately following treatment for 15min, and western blotted for total or active (phospho-Akt^Ser473^) Akt. **(A)** Relative Akt activity (phospho-Akt/Akt) of MDA-MB-231 cells following RQC or individual Res, Quer, or Cat at the indicated concentrations. **(B)** Relative Akt activity of MDA-MB-231 cells following vehicle or 15μM quercetin. **(C)** Relative Akt activity of MDA-MB-435 cells following vehicle or 15μM quercetin. For **(B)** and **(C)**, representative western blots from 3 separate experiments are shown. Graphs show the analyses of the integrated densities of positive bands relative to vehicle, as quantified from image J analysis. An asterisk indicates statistical significance (*p≤*0.05) when compared to vehicle.

To determine the contribution of individual polyphenols to the effect of combined RQC on mTOR signaling, MDA-MB-231 metastatic BC cells were treated with resveratrol, quercetin, or catechin individually, or in combination, at 1, 3, 5, 9 and 15μM for 15 min. Western blots of cell lysates were performed to detect the activation (phosphorylation) of AMPK, Akt, and the mTOR downstream effectors p70S6K and 4EBP1. In the MDA-MB-231 metastatic breast cancer cell line, treatment with RQC or resveratrol at concentrations >3 μM, inhibited Akt activity by ~50%, while catechin alone reduced Akt activity by ~40% at 15μM. Individual quercetin appeared to be the most efficient polyphenol at Akt inhibition, by inducing a statistically significant ~70% inhibition of Akt ser^473^ phosphorylation compared to vehicle treatment at concentrations ≥5 μM (Fig 1A and 1B, Fig A in S1 File). Similarly, 15μM quercetin inhibited the Akt activity in another metastatic cancer cell line MDA-MB-435, by ~70% (Fig 1C).

**Fig 2:**
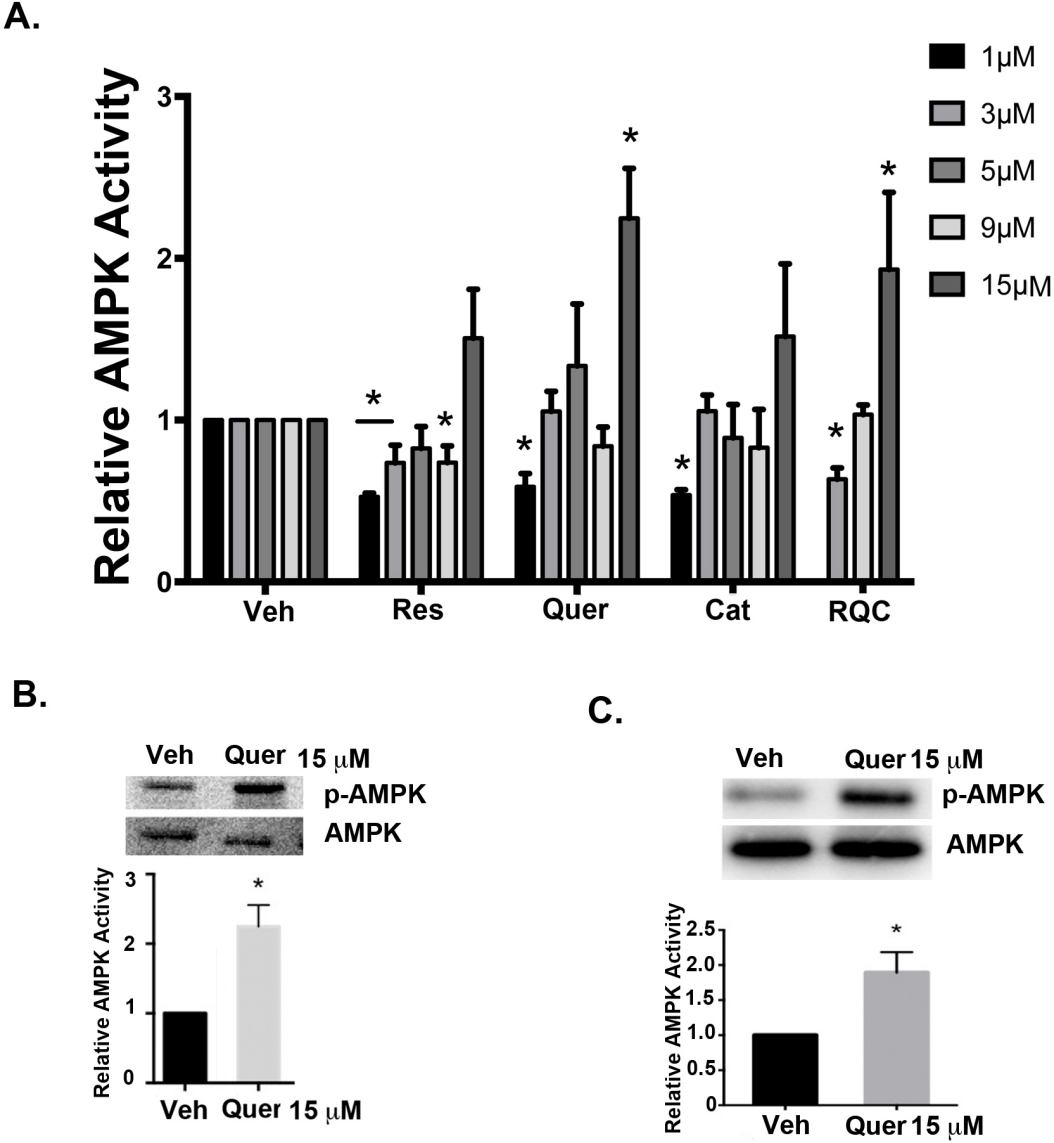
Effect of individual or combined RQC on AMPK activity in breast cancer cells. Quiescent MDA-MB-231 cells were treated with vehicle, or 1, 3, 5, 9, or 15μM of resveratrol (Res), quercetin (Quer), catechin (Cat), or combined RQC at 3μM total (1μM each), 9μM total (3μM each), or 15μM total (5μM each). MDA-MB-435 cells were treated only with vehicle and 15μM quercetin. Cells were treated for 15 min, lysed immediately, and western blotted for total or active (phospho-AMPK^Thr172^) AMPK. **(A)** Relative AMPK activity (phospho-AMPK/AMPK) of MDA-MB-231 cells following RQC or individual Res, Quer, or Cat at the indicated concentrations. Relative AMPK activity of **(B)** MDA-MB-231 cells following vehicle or 15μM quercetin, or **(C)** MDA-MB-435 cells following vehicle or 15μM quercetin. For **(B)** and **(C)**, representative western blots from 3 separate experiments are shown. Graphs show the analyses of the integrated densities of positive bands relative to vehicle as quantified from image J analysis. An asterisk indicates statistical significance (*p≤*0.05) when compared to vehicle.

The activity of AMPK, a negative regulator of mTOR signaling, increased by 2-fold when treated with quercetin at 15μM, in both MDA-MB-231 and MDA-MB-435 BC cells (Fig 2). In the MDA-MB-231 cell line, the increase in AMPK activity by quercetin at 15μM is similar to RQC at 5μM each (i.e. total polyphenol concentration of 15μM) indicating a concentration dependence of any polyphenol for the regulation of AMPK activity. Even though resveratrol and catechin at 15μM increased AMPK activity, this increase was not statistically significant. As reported previously [11], 5 μM individual compounds did not affect AMPK activity, while lower concentrations of all three compounds and the RQC combination significantly decreased AMPK activity (Fig 2A, Fig B in S1 File).

**Fig 3:**
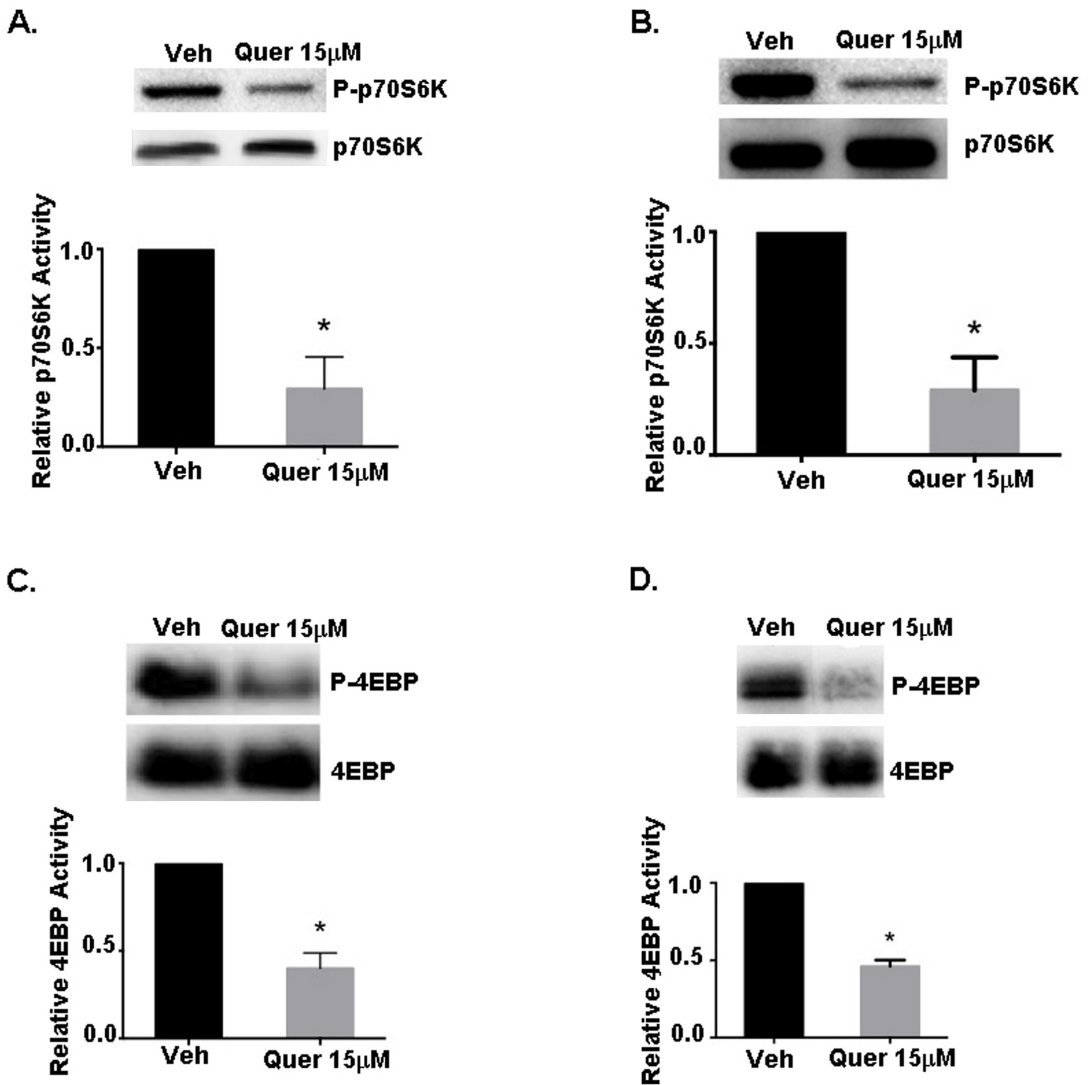
Effect of quercetin on mTOR signaling in breast cancer cells. Quiescent MDA-MB-231 and MDA-MB-435 cells were treated with vehicle or quercetin at 15μM for 15min, lysed immediately, and western blotted for total or active (phospho-p70S6K^Thr389^) p70S6K or (phospho-4EBP-1^Thr37/46^) 4EBP-1. **(A)** Relative p70S6K activity (phospho-p70S6K/p70S6K) from MDA-MB-231 cells. **(B)** Relative p70S6K activity from MDA-MB-435 cells. **(C)** Relative 4EBP-1 activity (phospho-4EBP-1/4EBP-1) from MDA-MB-231 cells. **(D)** Relative 4EBP-1 activity from MDA-MB-435 cells. Representative western blots from 3 separate experiments are shown. Graphs show the analyses of the integrated densities of positive bands relative to vehicle as quantified from image J analysis. An asterisk indicates statistical significance (*p≤*0.05) when compared to vehicle.

Since both Akt and AMPK activities can differentially regulate mTOR activity to control protein synthesis, we determined the effects of grape polyphenols on the activity of the mTOR downstream effector proteins p70S6K and 4EBP-1 by western blotting for phospho and active forms. Results show that quercetin at 15μM, reduced the phosphorylation of p70S6K by ~75% and 4E-BP1 by ~50% in both MDA-MB-231 and MDA-MB-435 BC cell lines (Fig 3). Decreased p70S6K and 4EBP phosphorylation is expected to result in inhibition of protein synthesis, since ribosome assembly requires the p70S6K substrate ribosomal S6 protein to be phosphorylated; and hyposphorylated 4EBP, the negative regulator of the eukaryotic initiation factor eIF4E, is expected to sequester eIF4E.

### Effects of quercetin and RQC on BC cell viability, apoptosis, and cell cycle progression

**Fig 4:**
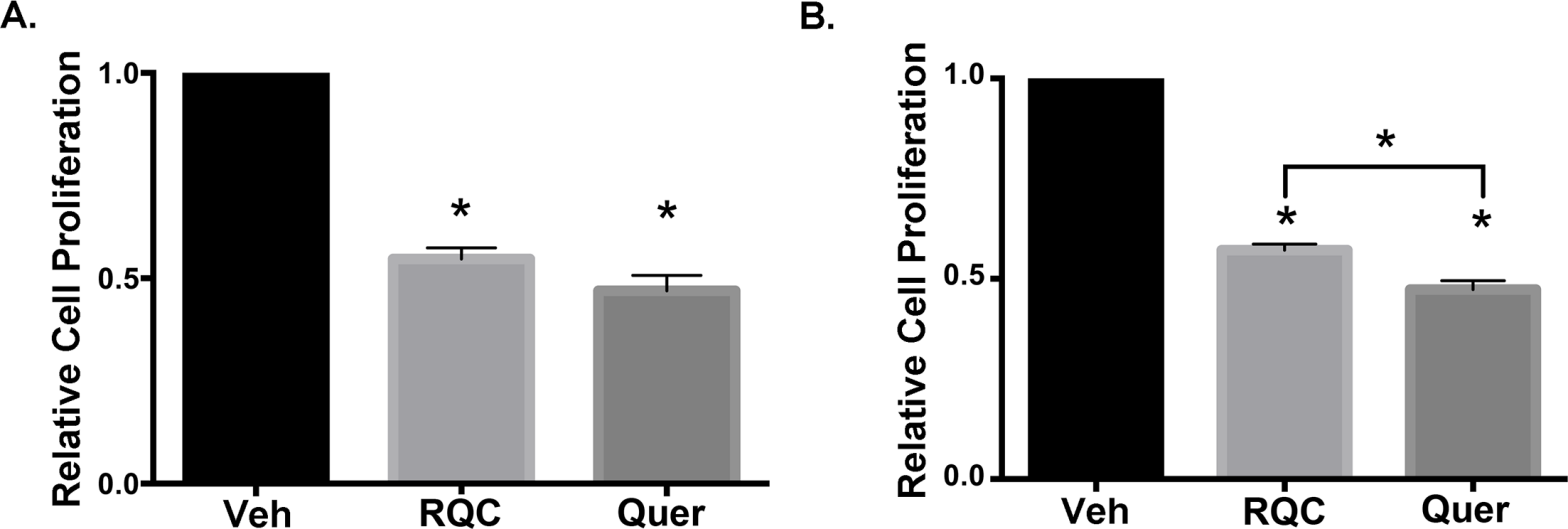
Effect of combined RQC or individual quercetin on breast cancer cell proliferation. MDA-MB-231 and MDA-MB-435 cells in 5% serum and phenol red-free media were treated with vehicle, combined resveratrol, quercetin, and catechin (RQC) at 5μM each or 15μM quercetin for 48h. Cells were fixed, nuclei stained with Propidium Iodide (PI), and intact (non-apoptotic) nuclei quantified. Percentage of viable cells ± SEM for 30 microscopic fields/triplicate treatments (N = 3) is presented. **A)** Average cell viability of MDA-MB-231 cells treated with RQC or quercetin relative to vehicle. **(B)** Average cell viability of MDA-MB-435 cells treated with RQC or quercetin relative to vehicle.

Since protein synthesis is essential for cell cycle progression, the decreased Akt and mTOR activities in response to quercetin are expected to affect BC cell viability and proliferation. Therefore, MDA-MB-231 and MDA-MB-435 BC cells were treated with 15μM quercetin or combined RQC polyphenols at 5μM each for 48hours, and tested for cell viability. Results show that in both MDA-MB-231 and MDA-MB-435 cells, RQC at 5μM each and quercetin at 15μM induced a ~50% decrease in cell proliferation (Fig 4). In the MDA-MB-435 cell line, quercetin at 15μM was significantly more inhibitory than RQC by demonstrating a 60% decrease in cell viability compared to the 40% decrease in response to RQC (Fig 4B).

**Fig 5:**
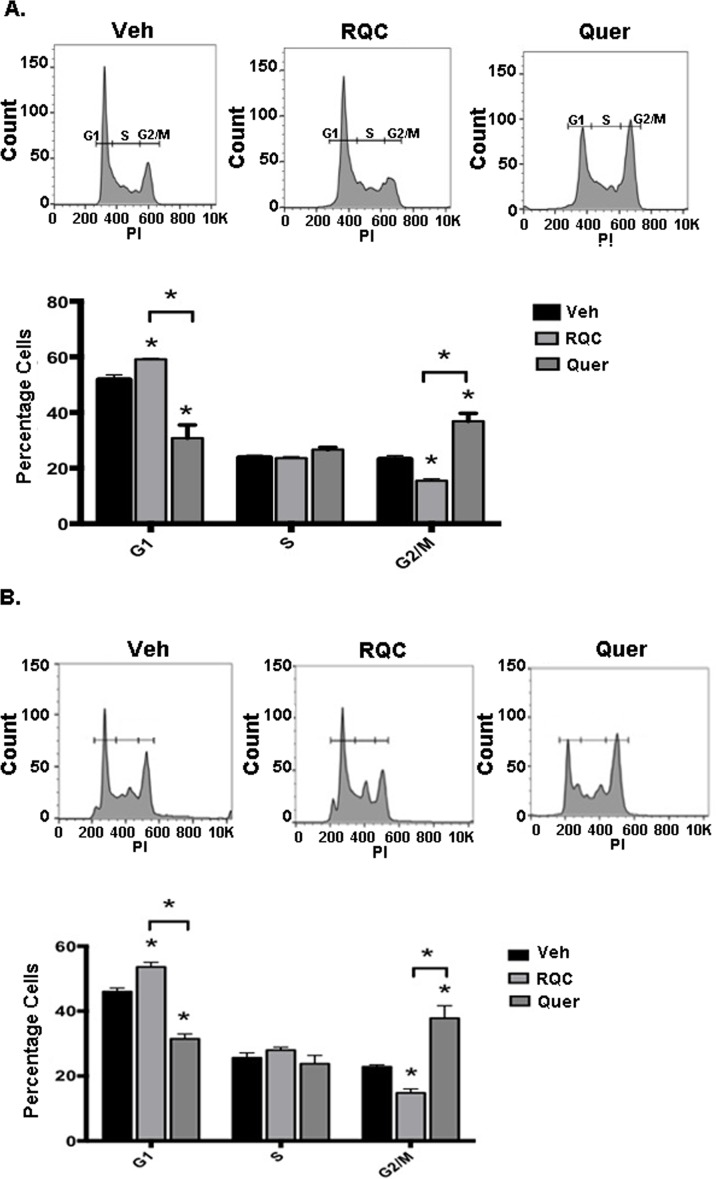
Effect of combined RQC or individual quercetin on the breast cancer cell cycle. Following treatment with vehicle, combined resveratrol, quercetin, and catechin (RQC) at 5μM each or 15μM quercetin for 48h, MDA-MB-231 or MDA-MB-435 cells were harvested, fixed in 70% ethanol, and stained with 40μg/ml PI. Cells were then analyzed using a FACS-Calibur instrument (Becton Dickinson, San Jose, CA). Samples from each treatment were gated similarly and the mean percentage of cells in the G0/G1, S, and G2/M phases quantified. **(A)** Upper panel: representative cell cycle histogram of MDA-MB-231 cells treated with grape polyphenols, lower panel: average cell percentage in each cell cycle stage. **(B)** Upper panel: representative cell cycle histogram of MDA-MB-435 cells treated with grape polyphenols, lower panel: average cell percentage in each cell cycle stage. N = 3, asterisk indicates statistical significance (*p≤*0.05) when compared to vehicle or between groups.

Due to the importance of cell cycle control in cancer progression, we tested the effects of combined RQC at 5μM each or quercetin at 15μM on cell cycle progression in MDA-MB-231 and MDA-MB-435 BC cells. Results show that as reported previously by us [24], the RQC treatment arrested the cell cycle in the G1 phase. However, quercetin treatment induced a G2/M phase cell cycle arrest in both cell lines, where 37% of cells were recovered in the G2/M phase following quercetin treatment, while only 23% and 15% of cells were in the G2/M phase from MDA-MB-231 or MDA-MB-435 cells treated with vehicle or RQC, respectively (Fig 5).

**Fig 6:**
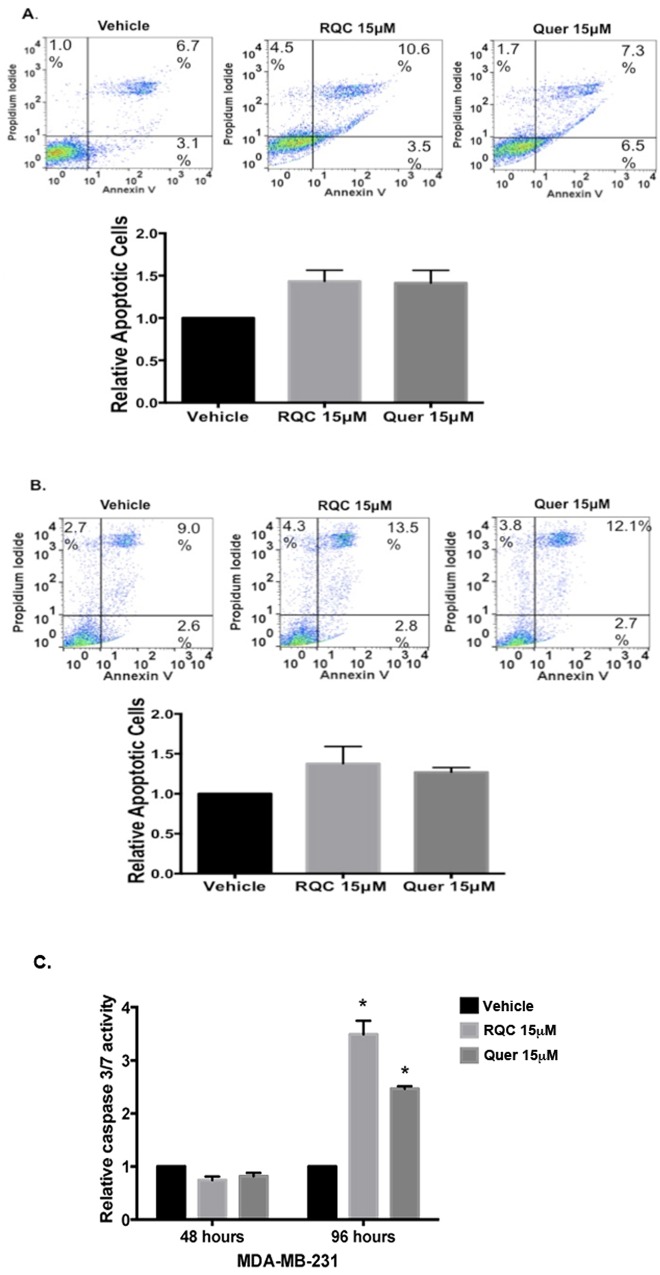
Effect of combined RQC or individual quercetin on breast cancer cell apoptosis. Quiescent MDA-MB-231 (**A**) or MDA-MB-435 (**B**) cells in 5% serum and phenol red-free media were treated with vehicle, combined RQC at 5μM each, or Quercetin 15μM for 48h. Trypsinized cells were incubated with Annexin V conjugate and propidium iodide, and analyzed by flow cytometry, using a four-color flow cytometer (FACSCalibur, BD Biosciences, San Jose, CA). Representative dot plots of 20,000 events/ treatment (N = 3), collected using Cell Quest software 3.3 (BD Biosciences, San Jose, CA) and analyzed using Flow Jo software vX.0.7 (BD Biosciences, San Jose, CA). Cell size and granularity were determined on forward versus side scatter (FSC vs. SSC). Annexin-V fluorescence was measured at 530/30 nm and Propidium Iodide at 585/42nm. The average percentage of early and late apoptotic cells obtained from Annexin V vs. Propidium Iodide dot plots are shown below. **(C)** Effect of combined RQC or individual quercetin on caspase 3/7 activity. Quiescent MDA-MB-231 cells in 96 well plate at 5% serum and phenol red-free media were treated with vehicle, combined RQC at 5μM each or quercetin 15μM for 48hr or 96hr. Then Caspase-Glo®3/7 reagent were added to each treatment, and after 1hr of incubation the luminescence was measured in a plate-reader luminometer. Relative luminescence to vehicle is shown, N = 3±SEM, asterisk indicates statistical significance (p≤0.05) when compared to vehicle.

To determine the mechanism by which quercetin and RQC reduces BC cell proliferation, we tested the effects of these compounds on apoptosis. Flow cytometry analysis of annexin V and PI stained MDA-MB-231 or MDA-MB-435 cells at 48h following RQC or quercetin at 15μM demonstrated 1.3–1.5-fold increases in the apoptotic cell population compared to vehicle controls (Fig 6A). Since these increases were not statistically significant, the activities of the effector caspase 3/7 were determined at 48h and 96h. We found that in the MDA-MB-231 cell line, caspase 3/7 activity was unchanged at 48 h following quercetin or RQC but was significantly increased following both RQC (3.5-fold) and quercetin (2.5-fold) treatments (Fig 6B).

Since, inhibition of mTOR and activation of AMPK have been implicated with autophagy induction, the effect of RQC or quercetin at 15μM for 48h, was also investigated in MDA-MB-231 or MDA-MB-435 cells by western blotting cell lysates for markers of the autophagy signaling pathway. However, the levels of autophagy related proteins did not change significantly following RQC or quercetin (Fig C in S1 File).

### Effects of quercetin and RQC on BC cell migration

**Fig 7:**
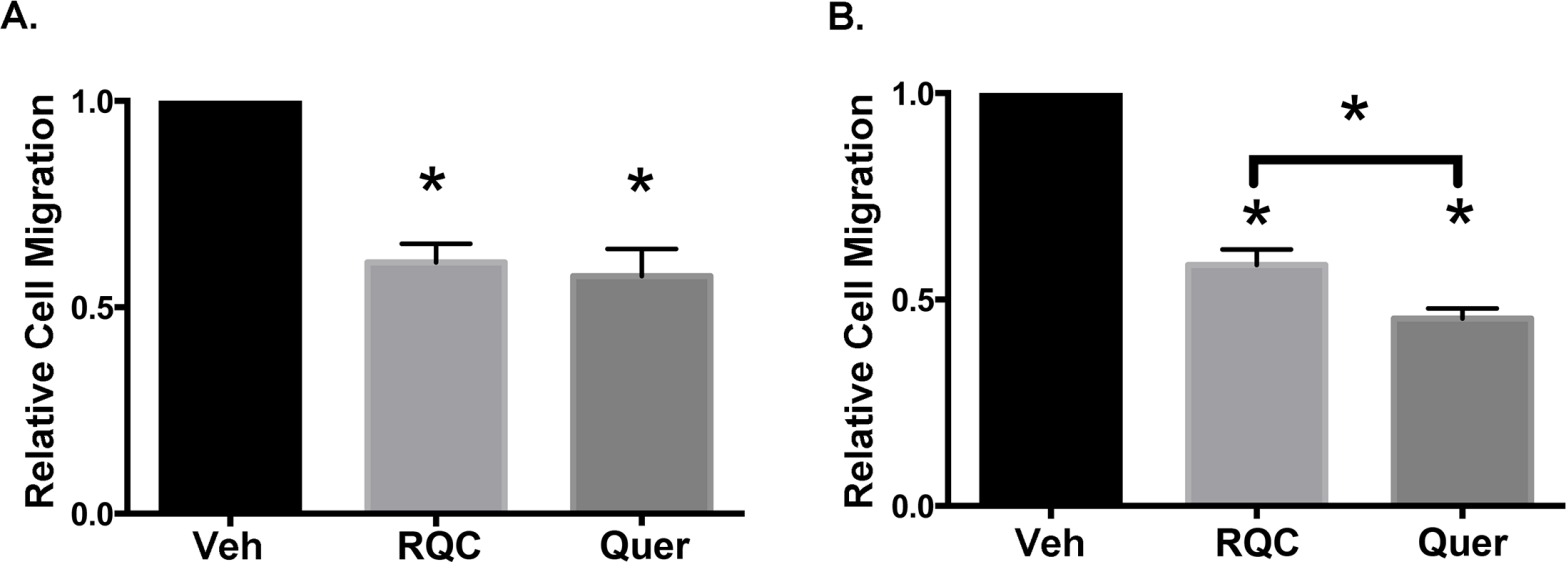
Effect of combined RQC or individual quercetin on breast cancer cell migration. Quiescent MDA-MB-231 and MDA-MB-435 cells were placed in the top of a transwell chamber with the bottom well containing: vehicle, combined resveratrol, quercetin, and catechin (RQC) at 5μM each, or 15μM quercetin in serum- and phenol red- free media. After 8h incubation, cells that migrated through the 8μM pore membrane were fixed, nuclei stained with Propidium Iodide and quantified. Percentage of migrated cells ± SEM for 15 microscopic fields/duplicate treatments for 3 independent experiments is presented. (**A)** Average cell migration of MDA-MB-231 cells treated with RQC or quercetin relative to vehicle. **(B)** Average cell migration of MDA-MB-435 cells treated with RQC or quercetin relative to vehicle.

Cell migration is central for development, tissue formation, and wound healing, and is considered to precede metastasis. Therefore, we tested the effect of quercetin at 15μM or RQC at 5μM each, in a Transwell assay, where the migration efficiency of control or treated BC cells was assessed by quantification of the number cells that migrated through 8μ pores of the transwell membrane. Treatment with either RQC or quercetin at 15μM resulted in a 40% inhibition of MDA-MB-231 cell migration (Fig 7A). For the MDA-MB-435 cells, there was a significant difference between a 60% inhibition of migration in response to quercetin (15μM) vs a 40% inhibition of migration in response to RQC (15μM) (Fig 7B). Thus, quercetin is a similar or more efficient inhibitor of BC cell migration when compared to RQC.

### Effects of quercetin on mammary tumor xenografts in SCID mice

**Fig 8:**
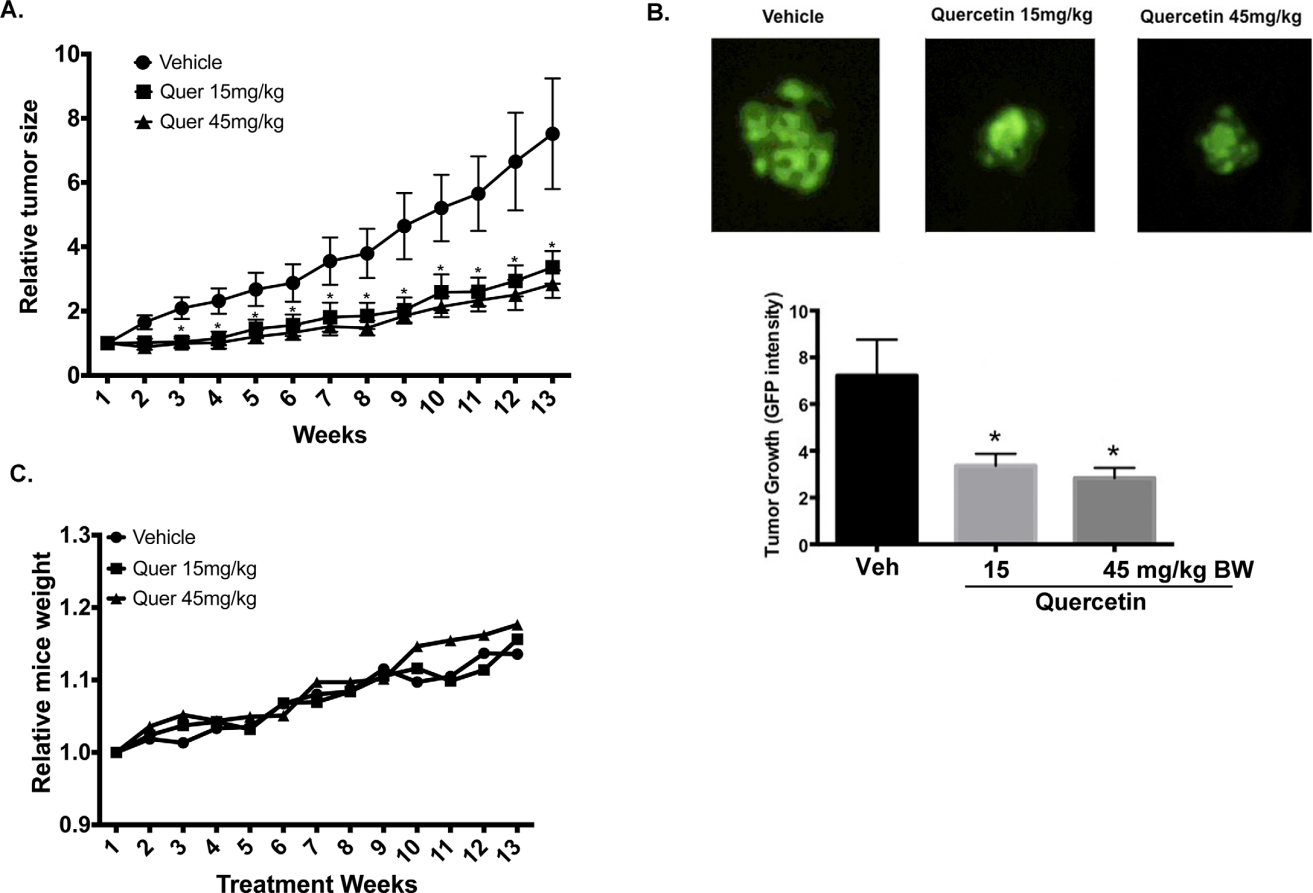
Effect of quercetin on the growth of MDA-MB-231 mammary fat pad tumors. GFP-MDA-MB-231 cells (0.5x10^6^) were inoculated at the mammary fat pad of SCID mice. One week after inoculation, mice were treated with vehicle, or quercetin at 15 or 45mg/kg BW three times a week by intraperitoneal injection. Fluorescence images of the mammary fatpad tumors were acquired once a week. **(A)** Average relative tumor area (N = 12) as a function of days following injection. Relative tumor area was calculated as the area of fluorescence, measured by fluorescence intensity on each day of imaging as a function of the fluorescence intensity of the same tumor on day 1. **(B)** Representative digital images of MDA-MB-231 tumors following vehicle, or quercetin at 15 or 45mg/kg BW at 13 weeks, followed by the quantification of tumor growth. N = 12±SEM, asterisk indicates statistical significance (*p≤*0.05) when compared to vehicle. **(C)** Relative SCID mice weight following vehicle, or quercetin at 15 or 45mg/kg BW treatments.

Since quercetin appeared to be a potent inhibitor of BC cell functions *in vitro*, we determined the effect of quercetin *in vivo*. To achieve this objective, female SCID mice were injected at the mammary fat pad with GFP-MDA-MB-231 BC cells, as described in [11]. After one week, mice were administered vehicle or quercetin at 15 or 45mg/kg BW three times per week for 13 weeks. As described in [28], the use of GFP-tagged BC cells, enabled the quantitative assessment of tumor growth via fluorescence *in situ* image analysis. Results show that quercetin treatment induced a significant reduction in tumor growth by ~70% compared to vehicle controls (Fig 8A and 8B). In order to assess toxicity, mice were weighted every week and no difference was observed between groups (Fig 8C). However, the higher quercetin concentration (45 mg/kg BW) resulted in severe constipation. Therefore, quercetin at 15mg/kg BW appears to be a physiologically relevant, non-toxic concentration for BC therapy.

## Discussion

Quercetin is the most prevalent flavonoid in the western diet [31], with an estimated daily intake of 15–40mg/kg [31,32], of which, 30–50% is absorbed [32]. After absorption, quercetin is deconjugated by β-glycosidase to the aglycone quercetin. During first pass metabolism, aglycone quercetin is metabolized to the methylated, sulphated or glucoronidated forms by enterocytic transferases [32–34]. Because of these conjugations, it is difficult to detect the aglycone form of quercetin in plasma, but considerable amounts still exist within tissues. This is because polyphenol conjugations can be hydrolyzed at the vascular level, yielding the aglycone forms in the tissues [35]. The maximum plasma concentration of quercetin is reached 9h after ingestion of a quercetin-containing food [34,36]. The administration of a dietary supplement of quercetin of 1–2g to mice resulted in a plasma concentration of 50 μM with an elimination half life of 25h [32]. Therefore, the 15μM concentration of quercetin used in the present study is achievable following dietary consumption of quercetin-rich foods or as a nutraceutical. Moreover, most of our experiments were conducted 24 or 48h following quercetin, which falls within the 24h elimination half-life for this polyphenol. The slow elimination of quercetin has been contributed to its particularly high affinity for plasma albumin [34]. Taken together, the concentrations and the incubation times used in our study are physiologically relevant, and indicate a viable anticancer role for quercetin.

Several previous studies using cell lines from diverse tumor types such as prostate, breast, cervical, ovarian, colon, liver, gastric, and lung have demonstrated an anticancer role for quercetin [32] Quercetin has been shown to suppress the tumorigenic activity of different carcinogens in colon cancer, downregulate PI3-K and protein kinase C, increase p53 levels arresting the cell cycle, and induce apoptosis of several cancer cell types, as demonstrated by: morphological alterations and DNA fragmentation, activation of caspases-3, 7 and 9, cleavage of PARP, and release of Cytochrome c, causing the activation of the mitochondrial pathway of apoptosis [37–40]. However, unlike in our study, where we did not see significant effects on apoptosis at 15 μM quercetin, most of these studies used quercetin at concentrations >25 μM, which are difficult to achieve via consumption of quercetin containing foods. A study that tested a number of cancer cell lines, including the estrogen receptor (ER) positive (+) MCF-7 breast cancer cell line, reported that quercetin at low concentrations (2μM) decreased the activity of 16 kinases, including those that control mitosis [41]. Quercetin was also shown to downregulate the transcription factor Twist to affect apoptosis in MCF-7 cells without affecting the viability of the MDA-MB-231 ER (-) breast cancer cell line, which was used in our study [40]. Therefore, the cellular effects of quercetin appear to be concentration- and cell type-dependent.

Of the RQC formulation, quercetin appears to be the most effective anticancer agent. Therefore, quercetin has been tested in phase 1 clinical trials in humans, and demonstrated antitumor activity when administrated 60–1400 mg/m^2^/wk by infusion in multi organ cancer patients, but dose-limiting nephrotoxicity was also described [37;42]. In another study, quercetin reduced the serum concentration of CA125 (ovarian cancer protein marker) by six-fold, and also decreased serum α-fetoprotein (hepatic tumor marker) [32]. These studies support the use of quercetin for cancer therapy, but quercetin has yet to be tested as an anti BC agent in clinical trials.

We previously reported that a combination of grape polyphenols (RQC at 5μM each) was more efficient than individual compounds at 5μM at inhibiting Akt and mTOR activities, cell proliferation, cell migration, cell cycle progression and tumor growth in the MDA-MB-231 human metastatic BC cell line. The previous study only tested the effect of quercetin at 5μM on Akt, AMPK, mTOR activities [11]. The objective of the present study is to demonstrate that quercetin alone, at similar concentrations to the combined RQC, can inhibit metastatic BC cells to comparable levels, and is the most active ingredient in the RQC formulation. We have extended the previous study to establish quercetin at 15μM as a potent inhibitor of Akt and mTOR activities leading to reduced cell proliferation, cell cycle arrest, apoptosis, and decreased cell migration. These results suggest that even though the three compounds in the RQC formulation work together at 5 μM each, potentially acting via the same pathway, quercetin contributes significantly to this inhibitory effect. This may be attributed to the fact that quercetin is an efficient inhibitor of PI3-K, the effector of Akt activity, with an IC_50_ ≈ 3.8μM [26] in comparison with resveratrol (one of the components of RQC) that has an IC_50_ ≈ 25μM [27].

We show that quercetin at 15μM, or RQC at 5μM each, exerted a similar effect on AMPK phosphorylation by a ~2 fold increase, suggesting a similar mechanism of AMPK activation for all three polyphenols. These results also suggest that RQC regulation of AMPK activity is concentration dependent, as AMPK activity was not affected by 3μM each RQC, but was activated 2-fold by 5μM each RQC. We also found a similar effect of quercetin in Akt/mTOR inhibition and AMPK activation in the triple negative MDA-MB-231 and HER2 positive MDA-MB-435 cells, suggesting that quercetin is effective in diverse BC subtypes. The regulation of AMPK by red wine polyphenols, and particularly quercetin, may be via regulation of the AMPK upstream effector kinases LKB1 and (or) calcium/calmodulin dependent kinase (CAMKK2), or the ATP/AMP ratio [43]. However, the specific mechanism remains to be determined.

Other groups have reported an inhibition of the mTOR pathway and activation of AMPK by quercetin at concentrations ≥ 25μM in prostate, colon, and other BC cell lines [44], Our studies show inhibition of Akt/mTOR activities by quercetin treatment at a lower concentration (15μM) that is closer to the physiological levels achieved after ingestion of a quercetin supplement. Therefore, quercetin appears to be the most effective component of RQC, and the observed regulation of cancer cell metabolism by quercetin has the potential to block BC cell growth, migration, and thus, metastatic progression.

Cell proliferation and increased cell survival are important factors in carcinogenesis, where cells with DNA damage and mutations will continue to grow [45]. Quercetin has been shown to have antiproliferative effects and to induce cell cycle arrest in liver, colon, pancreas, stomach, bladder, ovarian, and BC cells [46,47]. Accordingly, we found that both quercetin at 15μM and RQC at 5μM each, inhibits the triple negative MDA-MB-231 cell proliferation by ~40%, suggesting that quercetin can be substituted for RQC as an inhibitor of BC cell proliferation. The significant difference in the response of the MDA-MB-435 cell line between quercetin and RQC, where quercetin was a more efficient inhibitor of cell viability, as well as cell migration in MDA-MB-435 cells compared to the MDA-MB-231 cells, may be due to the more aggressive nature of the MDA-MB-435 cell line or the dependence on HER2/PI3K/Akt signaling for the survival of this HER2 overexpressing cell line.

Herein, we report an arrest in the G1 phase for the RQC treatment, and a G2/M arrest for the quercetin treatment in both cell lines. This discrepancy may be due to the resveratrol in the RQC formulation, which has been shown to induce Go/G1 arrest [48]. The observed cell cycle arrest at different phases could be due to the inhibition of different cell cycle checkpoint proteins by the different polyphenols. Our results are in agreement with a previous study that reported a quercetin-mediated cell cycle arrest in the G2/M phase in HeLa cells, due to regulation of Cyclin-D1 [49]. Since G2/M arrest can be due to a failure in protein synthesis during the S phase, quercetin may induce cell cycle arrest via decreased protein synthesis stemming from the Akt and mTOR inhibition.

Since cell cycle arrest is expected to precede apoptosis in most systems, and we have previously reported an increase in apoptosis in response to RQC [24], we investigated the effect of quercetin and RQC on apoptosis by two mechanisms. Early apoptosis was investigated by Annexin V staining at 48h; however, 48h incubation in quercetin, when the cells were arrested in the G2/M phase, did not demonstrate a statistically significant increase in apoptosis. As demonstrated by our previous study where the RQC formulation at 15μM did not affect apoptosis at 48h but did so at 96h following treatment [11], quercetin also induced apoptosis at 96h. Therefore, longer incubation times, or higher concentrations of quercetin, as was recently reported [40], may be required to induce apoptosis of breast cancer cells.

To investigate a potential role for quercetin in BC metastasis, we determined whether quercetin affects BC cell migration, the first step of cancer metastasis. Similar to our results with cell proliferation, quercetin exerted a more severe effect in the HER2 type MDA-MB-435 cell line, perhaps due to this cell line’s dependency on PI3-K signaling to regulate cell migration. Our data is in agreement with several studies that have correlated quercetin treatment with cell migration inhibition in cancers such as melanoma [50], teratocarcinoma [51], BC by inhibition of the PKCδ/ERK/AP-1 signaling [52], gliobastoma by IL-6 induction [53], and oral cancer by inhibition of MAPK, PI3K/Akt, Nf-κB, and uPA signaling [54]. *In vivo* analysis of quercetin treatment has also shown to be preventive of melanoma metastasis to lung [50]. Therefore, taken together, these results suggest that quercetin may act as a potent natural inhibitor of BC migration, and thus, metastasis, especially in HER2 type BC.

Finally, we tested an anticancer role for quercetin *in vivo*, using SCID mice with mammary tumor xenografts, and show that quercetin at 15 mg/kg reduced tumor growth by ~70%. This result compared to our previous data with the RQC formulation [11,25], suggests that quercetin is sufficient for the reduction of breast tumor growth *in vivo*. Even though no gross toxicity was observed, the mice receiving 45mg/kg quercetin demonstrated severe constipation. Since 15mg/kg BW quercetin demonstrated a similar inhibitory effect as 45mg/kg BW, we recommend using lower concentrations of quercetin. Therefore, ours is the first study to demonstrate the *in vivo* efficacy of quercetin at physiologically relevant concentrations. During the 13-week period of the present study, the mice did not form adequate metastases in the control treatments for statistical analysis. Therefore, future studies will also investigate the effect of quercetin on metastasis in the more aggressive MDA-MB-435 cell line.

In conclusion, quercetin at 15μM *in vitro* and 15 mg/kg BW *in vivo*, inhibits Akt/mTOR signaling, induces cell cycle arrest, and inhibits BC cell and tumor growth, and appears to be the most active ingredient in the RQC formulation. The inhibition of mTOR activities by quercetin via Akt inhibition and AMPK activation contributes to its anticancer effects. Overall, this study contributes to the mounting evidence of quercetin as a viable alternative therapeutic for BC that could potentially be used in combination with standard therapy for the reduction of toxic side effects of current chemo- and radio- therapeutic strategies.

## Supporting Information

S1 File Fig A. Effect of individual or combined RQC on Akt activity in breast cancer cells. Quiescent MDA-MB-231 cells were treated with (A) vehicle (V), combined Res, Quer, and Cat (RQC) at 3μM total (1μM each), or 1 μM of resveratrol (Res), quercetin (Quer), or catechin (Cat), (B) vehicle (V), 9μM total (3μM each) combined Res, Quer, and Cat (RQC), or 3 μM of resveratrol (Res), quercetin (Quer), or catechin (Cat), (C) vehicle (V) or 9μM of resveratrol (Res), quercetin (Quer), or catechin (Cat), or (D) vehicle (V), 15μM total (5μM each) combined Res, Quer, and Cat (RQC), or 15 μM of resveratrol (Res), quercetin (Quer), or catechin (Cat). Cells were lysed immediately following treatment for 15min, and western blotted for total or active (phospho-AktSer473) Akt. Each sub Figure (A, B, C, or D) shows a representative western blot and quantification of Relative Akt activity (phospho-Akt/Akt) from analyses of the integrated densities of positive bands relative to vehicle, as quantified from image J analysis. An asterisk indicates statistical significance (p≤0.05) when compared to vehicle. Fig B. Effect of individual or combined RQC on AMPK activity in breast cancer cells. Quiescent MDA-MB-231 cells were treated with (A) vehicle (V), combined Res, Quer, and Cat (RQC) at 3μM total (1μM each), or 1 μM of resveratrol (Res), quercetin (Quer), or catechin (Cat), (B) vehicle (V), 9μM total (3μM each) combined Res, Quer, and Cat (RQC), or 3 μM of resveratrol (Res), quercetin (Quer), or catechin (Cat), (C) vehicle (V) or 9μM of resveratrol (Res), quercetin (Quer), or catechin (Cat), or (D) vehicle (V), 15μM total (5μM each) combined Res, Quer, and Cat (RQC), or 15 μM of resveratrol (Res), quercetin (Quer), or catechin (Cat). Cells were lysed immediately following treatment for 15min, and western blotted for total or active (phospho-AMPK Thr172) AMPK. Each sub Figure (A, B, C, or D) shows a representative western blot and quantification of Relative AMPK activity (phospho-AMPK/AMPK) from analyses of the integrated densities of positive bands relative to vehicle, as quantified from image J analysis. An asterisk indicates statistical significance (p≤0.05) when compared to vehicle. Fig C. Effect of combined RQC or individual quercetin on breast cancer cell autophagy. Quiescent MDA-MB-231 and MDA-MB-435 cells in 5% serum and phenol red-free media were treated with vehicle, combined RQC at 5μM each, or Quercetin 15μM for 48h, lysed immediately and western blotted for protein autophagy markers (Beclin-1, ATG3, ATG5, ATG7 and ATG12). Representative western of N = 3 is shown.
(PDF)
