# Supplementary figures and images for: Anti-Breast Cancer Potential of Quercetin via the Akt/AMPK/Mammalian Target of Rapamycin (mTOR) Signaling Cascade
Source: PLoS One. 2016 Jun 10;11(6):e0157251. doi: 10.1371/journal.pone.0157251 (PMC4902235; doi:10.1371/journal.pone.0157251)

Supporting Information File 1

Figure A.

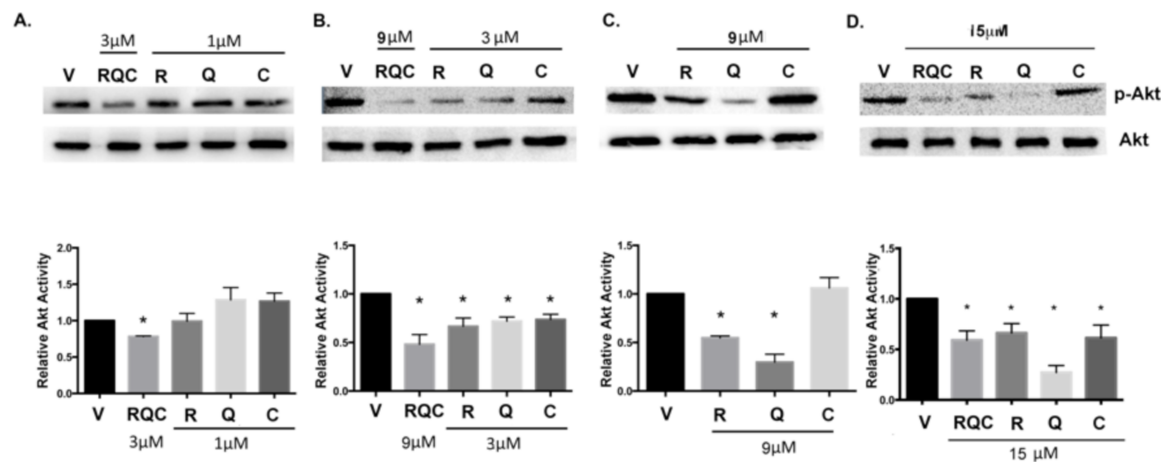

Figure B.

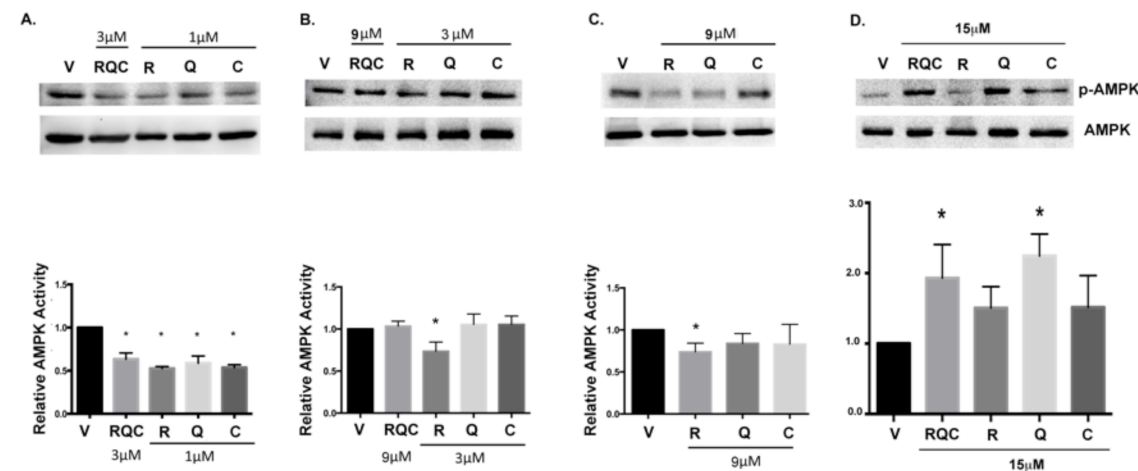

Figure C.

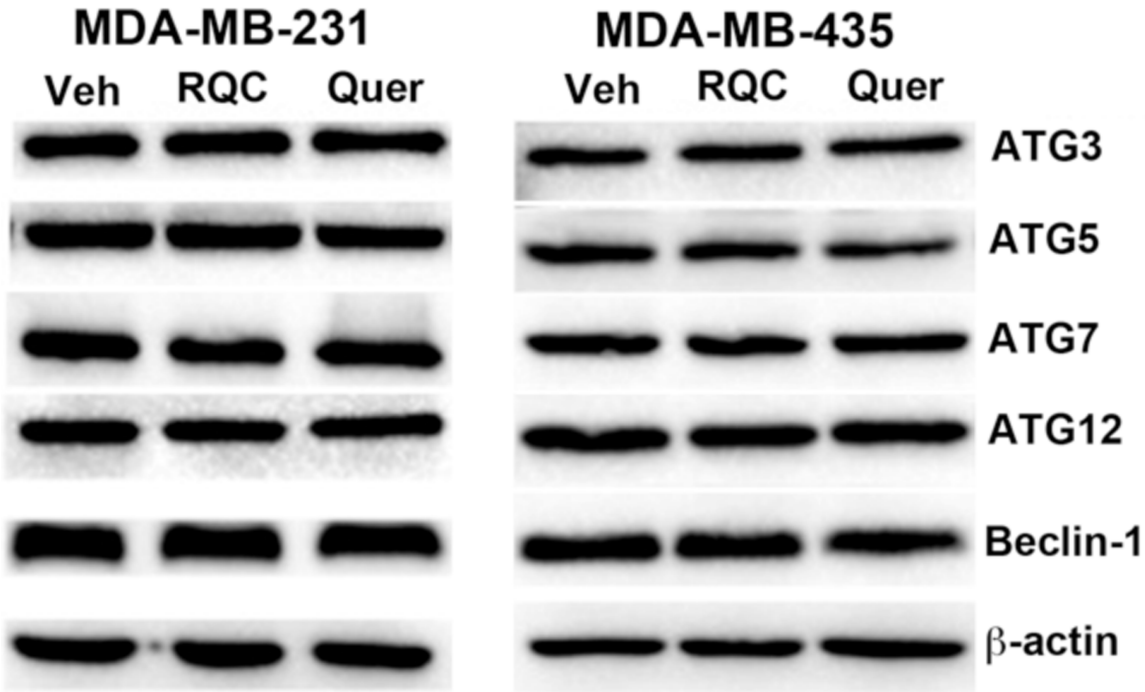

Supplement: S1 File — Fig A. Effect of individual or combined RQC on Akt activity in breast cancer cells. Quiescent MDA-MB-231 cells were treated with (A) vehicle (V), combined Res, Quer, and Cat (RQC) at 3μM total (1μM each), or 1 μM of resveratrol (Res), quercetin (Quer), or catechin (Cat), (B) vehicle (V), 9μM total (3μM each) combined Res, Quer, and Cat (RQC), or 3 μM of resveratrol (Res), quercetin (Quer), or catechin (Cat), (C) vehicle (V) or 9μM of resveratrol (Res), quercetin (Quer), or catechin (Cat), or (D) vehicle (V), 15μM total (5μM each) combined Res, Quer, and Cat (RQC), or 15 μM of resveratrol (Res), quercetin (Quer), or catechin (Cat). Cells were lysed immediately following treatment for 15min, and western blotted for total or active (phospho-AktSer473) Akt. Each sub Figure (A, B, C, or D) shows a representative western blot and quantification of Relative Akt activity (phospho-Akt/Akt) from analyses of the integrated densities of positive bands relative to vehicle, as quantified from image J analysis. An asterisk indicates statistical significance (p≤0.05) when compared to vehicle. Fig B. Effect of individual or combined RQC on AMPK activity in breast cancer cells. Quiescent MDA-MB-231 cells were treated with (A) vehicle (V), combined Res, Quer, and Cat (RQC) at 3μM total (1μM each), or 1 μM of resveratrol (Res), quercetin (Quer), or catechin (Cat), (B) vehicle (V), 9μM total (3μM each) combined Res, Quer, and Cat (RQC), or 3 μM of resveratrol (Res), quercetin (Quer), or catechin (Cat), (C) vehicle (V) or 9μM of resveratrol (Res), quercetin (Quer), or catechin (Cat), or (D) vehicle (V), 15μM total (5μM each) combined Res, Quer, and Cat (RQC), or 15 μM of resveratrol (Res), quercetin (Quer), or catechin (Cat). Cells were lysed immediately following treatment for 15min, and western blotted for total or active (phospho-AMPK Thr172) AMPK. Each sub Figure (A, B, C, or D) shows a representative western blot and quantification of Relative AMPK activity (phospho-AMPK/AMPK) from [file pone.0157251.s001.pdf]
